# Women’s satisfaction with intrapartum care in St Paul’s Hospital Millennium Medical College Addis Ababa Ethiopia: a cross sectional study

**DOI:** 10.1186/s12884-017-1428-z

**Published:** 2017-07-28

**Authors:** Tangute Demas, Tewodros Getinet, Delayehu Bekele, Teshome Gishu, Malede Birara, Yemesrach Abeje

**Affiliations:** 1 Department of Nursing, St. Paul’s Hospital Millennium Medical College, P.O Box 1271, Addis Ababa, Ethiopia; 2 Department of Public Health, St. Paul’s Hospital Millennium Medical College, P.O Box 1271, Addis Ababa, Ethiopia; 3 Department of Obstetrics & Gynecology, St. Paul’s Hospital Millennium Medical College, P.O Box 1271, Addis Ababa, Ethiopia

**Keywords:** Women, Satisfaction, Intrapartum care, Hospital, SPHMMC, Ethiopia

## Abstract

**Background:**

Satisfaction during intrapartum care is the most influential attribute on maternal health service return behaviors and utilization. Measuring satisfaction of women with intrapartum care helps to address the problems and improves the quality of delivery services. The aim of this study is to assess women’s level of satisfaction during intrapartum care.

**Method:**

A hospital based, analytic, cross sectional study was conducted at St. Paul’s Hospital Millennium Medical College (SPHMMC), Addis Ababa Ethiopia, from May to June 2015. Data collectors administered a structured and pretested questionnaire to collect data and then analyzed it using SPSS version 20.0 software.

Binary logistic regression was used to identify factors associated with women’s intrapartum care satisfaction.

**Result:**

A total of 394 women of mean age 25.98 years with a standard deviation of ±4.72were included in the study. Only 19% of the women were satisfied with the intrapartum care they received. The variables which were significantly associated with satisfaction of intrapartum care were; opportunity to talk Adjusted Odds Ratio (AOR) (95% CI) 2.44 (1.12, 5.29); Pain Management AOR (95% CI) 3.37 (1.83, 6.21); Short Length of Time Taken for Admission After Seen by Health Professionals AOR (95% CI)0 .97 (0.93, 0.99), and Short Length of Stay in the Hospital AOR (95% CI) 0.91 (0.87, 0.96).

**Conclusions:**

The women’s overall satisfaction with intrapartum care was low. Multiple factors influence their satisfaction. Health professionals, policy makers and health administrators should give emphasis to factors that contribute to low satisfaction of women with intrapartum care. They should also strengthen their efforts to deliver quality and easily accessible maternal health service to improve women’s overall satisfaction with the maternal health service.

**Electronic supplementary material:**

The online version of this article (doi:10.1186/s12884-017-1428-z) contains supplementary material, which is available to authorized users.

## Background

Patient satisfaction is a major component of quality of health care in service provision. Patient expectations of care and attitudes greatly contribute to satisfaction [1].Mostly; maternal satisfaction is determined by the physical environment of the health service, and the availability and accessibility of medicines and supplies. It is also affected by interpersonal communication with the health care provider, competency of the health care provider and support, and the health status of the mother and new born. [2]. Satisfaction during intrapartum care is the most influential attribute on service return behaviors and utilization [3].

Maternal health is closely associated with the strength of the health care system and the quality and accessibility of maternal health care services. Most maternal deaths occur during delivery and post delivery period [4].

Despite the fact that a higher maternal mortality ratio is seen in some countries, including Ethiopia, the number of mothers dying each year has been declining worldwide. In 2011, nearly two hundred fifty thousand maternal deaths occurred globally and. Only 23 countries accounted for 80% of the deaths. India, Nigeria, Indonesia, Ethiopia, Pakistan and Democratic Republic of Congo were in the first row [5].

Maternal mortality is high in Ethiopia due to delays in seeking health service, in reaching the health facility and in receiving timely and effective intervention after reaching the health facility [4]. The proportion of births attended by skilled providers is a measure of the health system’s effectiveness, accessibility and quality of care. However, in Ethiopia the proportion of births attended by a skilled birth attendant was 10% [6].

Even though health professionals are expected to provide holistic care that addresses the client’s physiological, psychological and emotional behaviors during the process of labor and birth [7], a study conducted in Addis Ababa, Ethiopia, on the status of respectful and non-abusive care during facility based child birth in a hospital and health centers revealed that the majority of the respondents faced disrespect and abuses [8].Another study on satisfaction with delivery service revealed that the majority of the women were not satisfied with procedures, explanation and information shared by health professionals and pain relief during labor [9].

Though delivery assisted by skilled providers is the most important proven intervention in reducing maternal mortality and one of the millennium development goals indicators to track national effort toward safe motherhood most women prefer home delivery because of perceived bad attitude and communication of the health care providers. Close relatives and traditional birth attendants who are nearby get more trust than health institutions [10, 11].

The World Health Organization (WHO) emphasizes ensuring patient satisfaction as a means of secondary prevention of maternal mortality, since satisfied women are more likely to adhere to health providers’ recommendations and utilization [1].

In some countries studies revealed that the quality of intrapartum care services from the perspective of clients was poor and women satisfaction was low. This is due to provider’s bad attitude and communication, longer duration of stay in the ward, inadequate information about the drug prescribed and explanation of procedures to be done to the client, experiencing an episiotomy and poor pain relief during labor and vaginal birth [10, 12–14].

In other studies, women’s level of satisfaction during labor and delivery was high. This is associated with wanted status of pregnancy, good maternal condition after delivery, short waiting time to see the health worker, availability of waiting area, provider’s measure taken to assure privacy during examinations and reasonable amount of cost paid for the service [15–17]. Women’s experiences with health care providers and facilities influence their care seeking decisions [18]. In a study conducted on “why do women prefer home births in Ethiopia,” women indicated that the main reasons were poor quality of care and previous negative experience with health facilities [19].

The purpose of measuring satisfaction is to understand patients’ experience and response to health care service, to measure the quality of care received and to identify problem areas for intervention [20]. Women satisfaction with intrapartum care is the most influential attribute on service return behaviors and utilization. Measuring satisfaction of women with intrapartum care helps to address the problems for improving the quality of delivery services. Hence, the objective of this research was to assess women’s level of satisfaction with intrapartum care and associated factors in St. Paul’s Hospitals Millennium Medical College, Addis Ababa, Ethiopia.

## Methods

### Study area and period

The study was conducted at St. Paul’s Hospitals Millennium Medical College (SPHMMC) in Addis Ababa, Ethiopia. SPHMMC is one of the largest public hospitals in the country and provides academic, research and medical services. The hospital provides antenatal care and delivery service for more than 2500 and 8000 women per year respectively. The study was conducted from May to June 2015.

### Study design

A hospital based, analytic, cross sectional study was conducted to assess level and factors associated with women’s satisfaction with intrapartum care.

### Study population

All women who were admitted to the maternity ward of St. Paul’s hospital and gave birth from May to June 2015 were included in the study. Women who delivered at home and were admitted for retained placenta, post partum hemorrhage or other complications were excluded.

### Sample size and sampling technique

The maximum sample size for this study was obtained using single population proportion formula**:**

$$ \mathbf{n}=\frac{{\left(Z\alpha /2\right)}^2p\left(1-p\right)}{d^2} $$

The assumptions were *p* = 61.9% (taken from a study conducted on mothers satisfaction in delivery services at a maternity referral hospital in Amhara region Ethiopia [17]. The level of confidence 95%, (Zα/2 = 1.96), margin of error between the sample and the population 5%, and 10% was considered for non-response. A sample size of 361 was calculated and adding a non-response rate of 10% a final sample size of 397 was taken.

Consecutive series of women who received delivery service in the hospital and who met the inclusion criteria were included in the study till the calculated sample size was achieved.

### Study variables

The outcome variable was women’s satisfaction status with intrapartum care. The explanatory variables include socio-demographic characteristics and general experience of labor and child birth.

### Data collection procedures

The questionnaire for this study was adopted with some modification from a study conducted in Jordan [13]. It was first translated from English to Amharic and then retranslated back to English by a linguistic graduate to assure its consistency. Discussion was made by a group of health professionals (obstetricians, midwife, nurse and public health) in detail to relate the survey instrument with the local context and to make it easily understandable by the respondents. The questionnaire was pretested in twenty mothers who delivered in other similar setting (Yekatit 12 Hospital, Addis Ababa) in order to give the data collector practical exposure and to get feedback to improve the tool, which in turn played role in data quality.

The data collectors were four female nurses, who were not working in the study hospital in order to reduce service providers’ bias. Training regarding the objective of the study and ways of administering the questionnaire was given to the data collectors and supervisors before the actual data collection.

An exit interview was administered by the data collectors up on discharge from the hospital. The questionnaire was composed of three parts: socio-demographic section, obstetric history and 14 satisfaction related variables.

### Data quality assurance

The quality of data was assured through careful adaptation and pretesting of the questionnaire. Proper training of the data collectors and supervisors, close supervision of the data collection procedures and proper categorization and coding of the data were attentively followed. The collected data were checked for its accuracy and completeness on a daily basis. In addition, data cleaning and checking was done before analysis.

### Operational definitions

*Intrapartum* was defined as the period from the beginning of contractions that cause cervical dilation to the first 1 to 4 h after delivery of the new born and placenta**.**

*Intrapartum care* was all the care provided to a woman during labor and delivery by nurses and other health professional including psychological support, management of normal labor and delivery, and detection and management of complications.

*Health care provider*s in this study were nurses, midwifes, interns, OB/Gyn residents and obstetricians working in the labor ward.

*Illiterate (variable):* those who cannot read and write.

*Private (variable)*: women who are nongovernmental organization employee.

*Satisfaction* was assessed using 14 Likertscale questions (Cronbach’s alpha of 0.835) measuring women’s satisfaction with 5 items related to interpersonal care by the health care provider (Cronbach’s alpha of 0.911), 4 items related to information received and involvement in decision making (Cronbach’s alpha of 0.681) and 5 items related to physical birth environment (Cronbach’s alpha of 0.678). Participants were asked to rate their satisfaction with intrapartum care on a five point Likert scale of 1- strongly disagree to 5 - strongly agree. The cut off point for satisfaction was adopted form a study conducted in Jordan [13] and calculated using the total mean score plus one standard deviation. The cut-off point for this study was 4.20 (over all mean (3.61) + standard deviation (0.59)). A woman who scored a mean score of greater or equal to 4.20 in the 14 scale Likert scale questions was considered as satisfied and a mean score less than 4.20 was considered as not satisfied.

### Data analysis

The data was entered into SPSS version 20 statistical software packages for data cleaning and analysis.

Frequencies were done to describe the different participants’ characteristics and to estimate the overall women’s satisfaction with respect to the intrapartum care.

Simple binary logistic regression model was fitted for each of the independent variables. Variables with *p*-value less than or equal to 0.20 [21] in the simple binary logistic regression were selected for the multiple binary logistic regression. An Adjusted Odds Ratio (AOR) was reported as a measure of association and all variables with a *p*-value <0.05 were considered as statistically significant factors associated with intrapartum care satisfaction.

### Ethical approval and consent to participate

Ethical approval and clearance was obtained from St. Paul’s Hospital Millennium Medical College Institutional Review Board. There were no potential risks that may cause any harm in any form on the study subjects. After obtaining permission from the Review Board, participants were provided with information about the objectives and importance of the study. Since there is no intervention, no measurement and no sample were taken; informed verbal consent was obtained in advance from the study subjects. All information which was communicated with individual subjects or organizations was kept private and confidential. Coding and aggregate reporting were used to eliminate respondents’ identification and ensure anonymity.

## Result

Out of the total sample size (*n* = 397), 394 women completely answered the questionnaires. Three questionnaires were excluded for incompleteness, giving a response rate of 99%. The study participants have a mean age of 25.98 years with a standard deviation of ±4.72 years. Most of the study participants (89.8%) were married. Among the study participants, 54.6% of them were from Addis Ababa and 39.3% of them attended primary education. More than 50 % of the study participants are Orthodox religion followers. Housewife was the most common occupation (58.1%) [Table 1].

**Table 1:** Socio demographic characteristics of delivering mothers in St Paul’s Hospital Millennium Medical College, Addis Ababa, Ethiopia, May – June 2015 (*n* = 394)

Variable	Group	N	%
Occupation	Government	29	7.4
	Private	40	10.2
	Student	16	4.1
	Merchant	19	4.8
	Housewife	229	58.1
	Other	61	15.5
Residence	Addis Ababa	215	54.6
	Other	179	45.4
Marital Status	Single	30	7.6
	Married	354	89.8
	Separated	8	2
	Divorced	2	0.5
Educational Status	Illiterate	82	20.8
	Primary	155	39.3
	Secondary	86	21.8
	Other	71	18
Religion	Orthodox	227	57.6
	Muslim	104	26.4
	Protestant	60	15.2
	Other	3	0.8
Ethnicity	Oromo	152	38.6
	Amhara	118	29.9
	Other	124	31.5

Among the study participants, only 75 (19%) were found to be satisfied based on the 14 Likert scale questions with cut-off point ≥4.20. Most of the government employees 26(89.7%) were not satisfied. Only 16.2% of women living outside Addis Ababa were satisfied, which was relatively small compared to those living in Addis Ababa (21.4%). All of the separated women were not satisfied. The majority of married, 289 (81.4%), housewives, 180 (78.6%) and Addis Ababa residents, 169 (78.6, %) were not satisfied. Of women who attained primary education, 126(81.3%) were not satisfied. Among those women who completed secondary school, 24.4% of them were satisfied, which is high as compared to the others [Table 2].

**Table 2:** Prevalence of intrapartum care satisfaction within socio-demographic characteristics of women in St Paul’s Hospital Millennium Medical College Addis Ababa, Ethiopia, May–June 2015 (*n* = 394)

Variable Group		Satisfaction Status	
		Satisfied n (%)	Not Satisfied n (%)
Occupation	Government	3 (10.3)	26 (89.7)
	Private	8 (20)	32 (80)
	Student	5 (31.2)	11 (68.8)
	Merchant	9 (47.4)	10 (52.6)
	Housewife	49 (21.4)	180 (78.6)
	Other	1 (1.6)	60 (98.4)
Residence	A.A	46 (21.4)	169 (78.6)
	Other	29 (16.2)	150 (83.8)
Marital Status	Single	9 (30)	21 (70)
	Married	65 (18.4)	289 (81.6)
	Separated	0 (0)	8 (100)
	Divorced	1 (50)	1 (50)
Educational Status	Illiterate	13 (15.9)	69 (84.1)
	Primary	29 (18.7)	126 (81.3)
	Secondary	21 (24.4)	65 (75.6)
	Other	12 (16.9)	59 (83.1)
Ethnicity	Oromo	28 (18.4)	124 (81.6)
	Amhara	25 (21.2)	93 (78.8)
	Other	22 (17.7)	102 (82.3)

To identify factors affecting women satisfaction, binary logistic regression model was used, as the outcome variable of satisfaction is a dichotomous variable.

First, a simple binary logistic regression was fitted for each of the independent variables to select potential factors for the multiple binary logistic regression. Then, multiple binary logistic regression was fitted by incorporating all variables with *p*-value less than or equal to 0.20 in the simple binary logistic regression, as recommended by Hosmer&Lemshow [21]. Considering the rule of thumb for logistic regression, variables with small a number of success (< 10) in each category were not included in the model.

Based on the results of the simple binary logistic regression [Table 3], the variables Residence, Seek to Talk to Professionals, Opportunity to Talk, Labor as Expected, Privacy, Pain Management, Time of Admission and Length of Stay in the Hospital were found to be significantly associated with women’s satisfaction at 20% level of significance. Therefore, these variables were selected to be included in the multiple binary logistic regression.

**Table 3:** Possible factors and their association with women intrapartum care satisfaction simple binary logistic regression

Variable	Group	COR (95% CI)	*P*-Value
Residence	A.A	1.41 (0.84, 2.35)	0.192^*^
	Other	–	–
Educational Status			0 .503
	Illiterate	0.93 (0.39, 2.19)	0 .861
	Primary	1.13 (0.54, 2.37)	0.743
	Secondary	1.59(0.72, 3.51)	0.252
	Other	–	–
Ethnicity			0.769
	Oromo	1.05 (0.57, 1.94)	0.884
	Amhara	1.25 (0.66, 2.36)	0.499
	Other	–	–
Episiotomy	Yes	0.84 (0.51, 1.39)	0.501
	No	–	–
Seek to Talk to Professionals	Yes	0.39 (0.23, .66)	0.001^*^
	No	–	–
Opportunity to Talk	Yes	4.44 (2.6, 7.59)	0.000^*^
	No	–	–
Labor as Expected	Yes	0.38 (0.23, 0.63)	0.000^*^
	No	–	–
Complication	Yes	.87 (.52, 1.46)	0.597
	No	–	–
Pain Management	Yes	4.44 (2.6, 7.59)	0.000^*^
	No	–	–
Privacy	Yes	2.17 (1.23, 3.84)	0 .008^*^
	No	–	–
Age		1.01 (0.95, 1.06)	0.846
Parity		1.06 (0.85, 1.34)	0.598
Time to be Seen by Professional (hrs)		0.99 (0.95, 1.03)	0 .636
Time of Admission (hrs)		0.95 (0.94, 1.01)	0 .068^*^
Length of Stay (hrs)		0.90 (0.86, 0.94)	0 .000^*^

*Significant at 20% level of significance

In order to identify the factors associated with women’s satisfaction by controlling possible confounders and to obtain the Adjusted Odds Ratio as a measure of association, multiple binary logistic regression analysis was fitted by incorporating all variables that were found to be significant at 20% level of significance by the simple binary logistic regression.

The results of the multiple logistic regression analysis revealed that the variables Opportunity to Talk, Pain Management, Time of Admission and Length of Stay in the Hospital were significantly associated with women’s intrapartum care satisfaction at 5% level of significance [Table 4].

**Table 4:** Possible factors and their association with women intrapartum care satisfaction multiple binary logistic regression

Variable	Group	AOR (95% CI)	*P*-Value
Residence	Addis Ababa	1.07 (0.58, 1.96)	0.835
	Other	–	–
Seek to Talk to Professionals	Yes	0 .85 (0.39, 1.87)	0.686
	No	–	–
Opportunity to Talk	Yes	2.44 (1.12, 5.29)	0.025^*^
	No	–	–
Labor as Expected	Yes	0.54 (0.29, 1.0)	0.051
	No	–	–
Pain Management	Yes	3.37 (1.83, 6.21)	0 .000^*^
	No	–	–
Privacy	Yes	1.55 (0.77, 3.14)	0.224
	No	–	–
Time of Admission (hrs)		0.97 (0.93, 0.99)	0 .031^*^
Length of Stay (hrs)		0.91 (0.87, 0.96)	0.000^*^

*Significant at 5% level of significance

In the multiple regression analyses, the odds/chance of satisfaction for women who had an opportunity to talk with health care providers was 2.44 times higher than those who did not have an opportunity to talk. Thus, those who had an opportunity to talk were more likely to be satisfied.

The odds/chance of satisfaction for women who had pain management was 3.37 times higher than those who did not. That is, those who had pain management were more likely to be satisfied.

The length of time taken for admission after being seen by the health professional and length of stay in the hospital were significantly associated with women’s intrapartum care satisfaction. For a 1 h increase in time (hr) of admission, the odds/chance of satisfaction for women was 0.97 times higher. Hence, it revealed that when the time of admission increases, women were less likely to be satisfied. For a 1 h increase in length of stay (hrs) in the hospital, the odds/chance of satisfaction for women was 0 .91 times higher, and it indicated that when the length of stay in the hospital increases, women were less likely to be satisfied.

## Discussion

This study assessed the level of women’s satisfaction with intrapartum care and its associated factors. The study’s finding indicated that the overall level of women’s satisfaction was low. Only 19% of the study participants were found to be satisfied based on the 14 likert scale questions with cut-off point ≥4.20. This finding was similar with the study conducted in Jordan on women’s satisfaction with hospital based intrapartum care where overall about 17.8% of women were satisfied with intrapartum care [13]. The similarity of these findings may be due to the same status or level of quality maternal health service.

In contrast, the finding of this study was less than other studies findings which were conducted in Brazil and Serbian public hospitals on women’s satisfaction with childbirth care. Those studies revealed that a high proportion of women were satisfied (67%, 81%) respectively [15, 16]. Also, a study conducted in Amhara region, South Ethiopia and North west Ethiopia on satisfaction of mothers with referral hospitals’ delivery service revealed that the proportion of mothers who were satisfied with delivery care was high (61.9, 67.9 and 74.9%) respectively [17, 22, 23]. This might be because of different setting and study population background as our study was done in Addis Ababa, the capital city of Ethiopia. Their study was performed in three regional referral hospitals, where most women were from the rural area and whose knowledge and expectations about quality of care might be low. This possibility is further strengthened by the finding of a study conducted in Gandhi Memorial Hospital Addis Ababa, Ethiopia which revealed similar finding to our study with a satisfaction of less than 21%.

Different studies revealed that women’s satisfaction with delivery care was associated with proper pain management. A study conducted in Jordan on women satisfaction with hospital based intrapartum care revealed that low satisfaction was associated with poor pain relief during labor and vaginal birth [13]. A study conducted in Tabriz hospitals revealed that, lack of labor pain management and communication about the progress of labor caused the most dissatisfaction with the service [9]. A study assessing client satisfaction in labor and delivery services at a maternity referral hospital in Ethiopia also showed that 82% of clients were reporting dissatisfaction due to poor pain control methods [10]. In our study, both in simple and multiple analysis, pain management was found to be significantly associated with women’s satisfaction. These findings may indicate that laboring women needed special attention on pain management. The trend has been to consider labor pain as a normal physiologic phenomenon with no need of pain management. However, health professionals should give emphasis for labor pain and manage depending on the nature and severity of the pain.

A study conducted on assessment of client satisfaction in labor and delivery services at a maternity referral hospital in Ethiopia showed that waiting time to see the health worker and longer duration of stay in the ward were independent predictors of client satisfaction [10].It is similar with this research finding which shows that length of time to see health care providers after arrival of the hospital, length of time for admission after seen by physician and length of stay in the hospital were significantly associated with women’s satisfaction. These findings may remind us about the importance of implementing a balanced score card (a strategic planning and management system which is used for performance measurement) into the Ethiopian healthcare service delivery system. This system should aim to provide patient contact within a few minutes of newly registered cases by a healthcare provider or reduction in out-patient waiting time and a reduction in average length of stay in the hospital.

## Conclusion

Measuring women’s satisfaction with intrapartum care will help to understand women’s experiences and responses to intrapartum care, to measure the quality of care received and to identify problem areas. In this study, women’s overall satisfaction with intrapartum care was low. Multiple factors influence women’s satisfaction; the opportunity to talk, pain management, length of time for admission and length of stay in the hospital were found to be significantly associated with women’s satisfaction.

The extent to which health care users are satisfied with the available service may be a key factor affecting their health behavior and health care utilization. Considering this, health professionals, policy makers and health administrators should give emphasis to factors that contribute to low satisfaction of women during intrapartum care and strengthen their efforts to deliver quality and easily accessible maternal health service to improve women’s overall satisfaction with the maternal health service.

Health care professionals and other caregivers should establish a rapport with the laboring woman. They should ask about her wants and expectations for labor in a respectful manner. The information provided will support and guide her through her labor.

This study also strongly suggested to the study hospital to work hard to expand the services for child birth by improving infrastructure and trained human resource. The care should be client centered easily accessible and high quality to increase women’s levels of satisfaction. In addition, further research should be done at a community level to address women who did not come to health facilities and to see the problem from the health care providers’ perspective.

This study had some limitations. The measurement of women satisfaction relied on the self response of participants which may be liable to response bias. Data obtained in this study was from a single hospital, which affects the generalizability of the results.

## Additional file

Additional file 1: Questionnaire. (DOCX 17 kb)

## Abbreviations

AA: Addis Ababa
AOR: Adjusted odds ratio
COR: Crude odds ratio
Hrs: Hours
SPHMMC: St. Paul’s Hospital Millennium Medical College
WHO: World Health Organization

## References

[1] Morris BJ, Jahangir AA, Sethi MK. Patient satisfaction: an emerging health policy issue, AAOS Now. 2013;7(6):29.

[2] Srivastava A, Avan BI, Rajbangshi P, Bhattacharyya S (2015). Determinants of women’s satisfaction with maternal health care: a review of literature from developing countries. BMC Pregnancy Childbirth.

[3] National Institute for Health and Clinical Excellence, Intrapartum care: Care of healthy women and their babies during childbirth Clinical guideline [CG55] Published date: September 2007.

[4] Government of Federal Democratic Republic of Ethiopia, United Nations Country Team. In: Government of Federal Democratic Republic of Ethiopia, United Nations Country Team, editor. Assessing progress towards the Millennium Development Goals. Ethiopia MDGS Report 2012. Addis Ababa; 2012.

[5] United Nations. Accelerating progress in saving the lives of women and children. Global Campaign for the Health Millennium Development Goals – Report, January, 2013.

[6] Central Statistical Agency [Ethiopia] and ICF International. Ethiopia demographic and health survey 2011. Addis Ababa (ET): Central Statistical Agency; Calverton: ICF International; 2012.

[7] Rider M (2003). Integrating Rubin’s framework with social support theory: principle and practice. JOGNN.

[8] Asefa A, Bekele D (2015). Status of respectful and non-abusive care during facility-based childbirth in a hospital and health centers in Addis Ababa, Ethiopia. Reprod Health.

[9] Naghizadeh S, Kazemi AF, Ebrahimpour M, Eghdampour F (2013). Assessing the factors of mother’s dissatisfaction with labor and delivery care procedure in educational and non-educational hospitals in Tabriz. Eur J Exp Biol.

[10] Melese T, Gebrehiwot Y, Bisetegne D, Habte D (2014). Assessment of client satisfaction in labor and delivery services at a maternity referral hospital in Ethiopia. Pan African Med J.

[11] Birmeta K, Dibaba Y, Woldeyohannes D (2013). Determinants of maternal health care utilization in Holeta town, Central Ethiopia. BMC Health Serv Res.

[12] Kigenyi O, Tefera GB, Nabiwemba E, Orach CG. Quality of intrapartum care at Mulago national referral hospital, Uganda: clients’ perspective. BMC Pregnancy Childbirth. 2013;13:162. 10.1186/1471-2393-13-162 PMC3751160 23941203

[13] Mohammed K, Shaban I, Homer C, Creedy D (2014). Women’s satisfaction with hospital based intrapartum care: a Jordanian study. Int J Nurs Midwifery.

[14] Senarath U, Fernando DN, Rodrigo I (2006). Factors determining client satisfaction with hospital-based perinatal care in Sri Lanka. Tropical Med Int Health.

[15] Matejić B, Milićević MS, Vasić V, Djikanović B (2014). Maternal satisfaction with organized perinatal care in Serbian public hospitals. BMC Pregnancy Childbirth.

[16] Rosa D, Moreira MS (2004). Aspects of women’s satisfaction with childbirth care in a maternity hospital in Rio de Janeiro, Brazil. National Libr Med.

[17] Tayelgn A, Zegeye DT, Kebede Y (2011). Mothers’ satisfaction with referral hospital delivery service in Amhara Region, Ethiopia. BMC Pregnancy Childbirth.

[18] Shiferaw T, Berhane Y, Gulema H, Kendall T, Austin A (2016). A qualitative study on factors that influence women’s choice of delivery in health facilities in Addis Ababa Ethiopa. BMC Pregnancy Childbirth.

[19] Shiferaw S, Spigt M, Godefrooij M, Melkamu Y, Tekie M. Why do women prefer home births in Ethiopia? BMC Pregnancy Childbirth. 2013;13:5. 10.1186/1471-2393-13-5 PMC3562506 23324550

[20] Ware JE, Snyder MK, Wright WR, Davies AR (2003). Defining and measuring patient satisfaction with medical care. Eval Program Plann.

[21] Hosmer DW, Lemshow S (2000). Applied logistic regression.

[22] Moshago T, Darebo T, Abera M, Abdulahi M. Factors affecting with client satisfaction with institutional delivery at public health facilities in South Ethiopia. J Med Physiol Biophys. 2016; 25.

[23] Mekonnen ME, Yalew WA, Anteneh ZA (2015). Women’s satisfaction with child birth care in FelegeHiwot referral hospital Bahir Dar city North West Ethiopia 2014 cross sectional study. BMC Res Notes.

