# Supplementary material for: Women’s satisfaction with intrapartum care in St Paul’s Hospital Millennium Medical College Addis Ababa Ethiopia: a cross sectional study
Source: BMC Pregnancy Childbirth. 2017 Jul 28;17:253. doi: 10.1186/s12884-017-1428-z (PMC5534094; doi:10.1186/s12884-017-1428-z)
Supplement: Additional file 1: — Questionnaire. (DOCX 17 kb) [file 12884_2017_1428_MOESM1_ESM.docx]

This is the questionnaire which is taken from (Mohammed K, Shaban I,Homer C, Creedy D. Women’s satisfaction with hospital based intrapartum care: a Jordanian study. International Journal of Nursing and Midwifery.2014; 6:32-39) and modified.

**. Part 1.Questions concerning labour and delivery**

| S.NO | **Questions** | **Possible response** |
| --- | --- | --- |
| 2.1 | Did you plan to conceive this pregnancy? | Yes---------------------------------------------------------1  No----------------------------------------------------------2 |
| 2.2 | How many deliveries have you had? | --------------------------------------------------------------- |
| 2.3 | How did your labour started | Spontaneous onset----------------------------------------1  By medication---------------------------------------------2 |
| 2.4 | How long did you wait to be seen by health professional after arriving to this hospital? | ---------------------------------------------------------- |
| 2.5 | How long did you wait for admission after you have been seen by the health professional | -------------------------------------------------------------- |
| 2.6 | What type of delivery you had? | Spontaneous vaginal delivery---------------------------1  Forceps/vaccum------------------------------------------2  Caesarean section----------------------------------------3 |
| 2.7 | Have you had an episiotomy during your current delivery? | Yes---------------------------------------------------------1  No----------------------------------------------------------2 |
| 2.8 | During your current delivery did you seek to talk with health professional about your labour/ birth? | Yes---------------------------------------------------------1  No----------------------------------------------------------2 |
| 2.9 | If your answer for question 2.8 is yes have you got a health professional to talk with about your labour and/or birth | Yes---------------------------------------------------------1  No----------------------------------------------------------2 |
| 2.10 | When you talk with the health care provider was the communication understandable/with your own language/ | Yes---------------------------------------------------------1  No----------------------------------------------------------2 |
| 2.11 | Who attended your delivery? | Midwife ---------------------------------------------------1  Nurse-------------------------------------------------------2  Intern-------------------------------------------------------3  Resident ---------------------------------------------------4  Obstetrician-----------------------------------------------5  I Don’t know -------------------------------------------6 |
| 2.12 | What did you think about the number of staffs during delivery around you? | Too many hospital staff around me--------------------1  About the right number of hospital staff around-----2  Too few hospital staff around---------------------------3 |
| 2.13 | How long did you stay in hospital during labour and birth? | ---------------------------------------------------------------- |
| 2.14 | Did your labour complete as expected? | Yes---------------------------------------------------------1  No----------------------------------------------------------2 |
| 2.15 | Did you have any complication during your current labor? | Yes---------------------------------------------------------1  No----------------------------------------------------------2 |
| 2.16 | If yes for question2.7, What type of complication did you have? | PPH---------------------------------------------------------1  Obstructed labor------------------------------------------2  Fetal distress----------------------------------------------3  Other ------------------------------------------------------4 |
| 2.17 | Did you get antipain when you were in labour? | Yes---------------------------------------------------------1  No----------------------------------------------------------2 |
| 2.18 | During labour and birth did the health care provider provided you adequate privacy? | Yes---------------------------------------------------------1  No----------------------------------------------------------2 |

**PART II- Questions concerning health care provider client interaction during labour and delivery.**

| **S.No** | **Questions** | **Possible responses** | | | | |
| --- | --- | --- | --- | --- | --- | --- |
|  |  | **Strongly agree=5** | **Agree**  **=4** | **Don’ t Know=3** | **Disagree**  **=2** | **Strongly disagree =1** |
| **3.1** | **Interpersonal care** |  |  |  |  |  |
| 3.1.1 | Health care provider were friendly and welcoming when you arrived at the hospital |  |  |  |  |  |
| 3.1.2 | Health care providers were encouraging and reassuring during labour and/or birth. |  |  |  |  |  |
| 3.1.3 | Health care providers were helpful during labour and/or birth. |  |  |  |  |  |
| 3.1.4 | During labour and/or birth physicians were more helpful. |  |  |  |  |  |
| 3.1.5 | The overall care during labour and birth was good |  |  |  |  |  |
| **3.2** | **Information provision &decision making** |  |  |  |  |  |
| 3.2.1 | The health care provider always kept you informed about what was happening during labour &/ or birth.. |  |  |  |  |  |
| 3.2.2 | During labour and/or birth decisions made without taking your wishes in to account |  |  |  |  |  |
| 3.2.3 | You felt pressured to have the baby quickly |  |  |  |  |  |
| 3.2.4 | You felt labour was taken over by strangers and/or machines |  |  |  |  |  |
|  | . **Physical birth environment** |  |  |  |  |  |
| 4.1 | During labour&/or birth the level of light was adequate |  |  |  |  |  |
| 4.2 | During labour&/or birth the room was spacious and adequate for your need |  |  |  |  |  |
| 4.3 | During labour&/or birth the level of noise was appropriate |  |  |  |  |  |
| 4.4 | During labour&/or birth trays and other equipments were clean |  |  |  |  |  |
| 4.5 | During labour&/or birth you were able to find the supplies that you needed |  |  |  |  |  |
